# Serum Concentrations of Betatrophin and Its Association with Indirect Indices of Insulin Resistance and Beta Cell Function in Women with Polycystic Ovary Syndrome

**DOI:** 10.1155/2017/2316986

**Published:** 2017-06-18

**Authors:** Agnieszka Adamska, Agnieszka Łebkowska, Małgorzata Jacewicz, Anna Krentowska, Justyna Hryniewicka, Sławomir Wołczyński, Maria Górska, Irina Kowalska

**Affiliations:** ^1^Department of Endocrinology, Diabetology and Internal Medicine, Medical University of Białystok, Białystok, Poland; ^2^Department of Reproduction and Gynecological Endocrinology, Medical University of Białystok, Białystok, Poland

## Abstract

**Introduction:**

Data underline the role of betatrophin in glucose homeostasis. Polycystic ovary syndrome (PCOS) is characterized by insulin resistance (IR). The aim of our study was to investigate the relationship of serum betatrophin concentrations with indirect indices of IR and insulin secretion in women with PCOS, compared to the control group.

**Methods:**

The study group comprised 43 women with PCOS and 16 controls. IR was assessed by HOMA-IR and Matsuda index. Insulin secretion was evaluated with HOMA-B. An oral glucose tolerance test (OGTT) with estimation of serum betatrophin concentrations was performed.

**Results:**

Glucose load resulted in an increase in serum betatrophin concentrations in the control group (*p* = 0.02). Serum betatrophin concentrations at 120 min of OGTT were lower in women with PCOS than in the control group (*p* = 0.02). We observed positive correlations between baseline serum betatrophin concentrations and HOMA-IR (*r* = 0.39, *p* = 0.008), negative correlations with Matsuda index (*r* = −0.31, *p* = 0.004), and a positive relationship with HOMA-B (*r* = 0.38, *p* = 0.01) in women with PCOS. Multiple regression analysis revealed that HOMA-B (*β* = 0.47, *p* = 0.001) was an independent factor connected to serum betatrophin levels in PCOS.

**Conclusions:**

Serum concentrations of betatrophin are connected with insulin resistance and beta cell function and did not change after glucose load in women with PCOS.

## 1. Introduction

Polycystic ovary syndrome (PCOS) is a frequent endocrinopathy which affects about 5–10% of reproductive-aged women [1]. Patients with PCOS may present a variety of reproductive and metabolic disturbances, including oligo/anovulation, polycystic ovary in the ultrasound, and hyperandrogenism, as well as insulin resistance [2]. Moreover, it has been shown that beta cell dysfunction is present in PCOS individuals [3]. It is widely known that insulin resistance and impaired beta cell function play a crucial role in the pathogenesis of type 2 diabetes mellitus (T2D). Therefore, women with PCOS, who often show both aforementioned traits, are at higher risk of impaired glucose tolerance, T2D, as well as disturbances in lipid profile [4].

Betatrophin, also called refeeding-induced fat and liver protein (RIFL) [5], lipasin [6], or atypical angiopoietin-like protein 8 (ANGPLT8) [7], is a 22 kDa hormone. In mice, betatrophin is produced by the liver, white adipose tissue (WAT), and brown adipose tissue (BAT), while in humans, it is mainly produced by the liver [6]. It has been shown that betatrophin has a dual role: it affects glucose homeostasis and lipid metabolism [8–12].

In a mouse model of insulin resistance induced by insulin receptor antagonist S961, hepatic and WAT overexpression of betatrophin was observed. This was accompanied by beta cell replication, followed by improved glucose tolerance [8]. The results obtained by these authors suggest that betatrophin can exert a promoting role on beta cell replication. However, these data were not confirmed by Gusarova et al. [13]. They reported that ANGPTL8^−/−^ mice with insulin resistance induced by a high-fat diet or insulin receptor antagonist S961 had normal beta cell expansion. This indicates that betatrophin is not necessary for beta cell proliferation. Interestingly, in their experiment, a significant reduction of triglyceride (TG) in ANGPTL8^−/−^ mice in comparison to wild-type mice was observed [13].

In humans, the role of betatrophin in glucose metabolism is unclear. Studies that investigated its action in both healthy subjects and patients with diabetes have brought conflicting results so far. Some researchers showed increased levels of serum betatrophin in T2D [14, 15], type 1 diabetes mellitus (T1D) [16], and gestational diabetes [17], whereas other data report decreased serum betatrophin concentrations in obese individuals and T2D patients [11].

Interestingly, it has also been demonstrated that there are no significant differences in serum betatrophin levels between nondiabetic and T2D patients and between the lean and the morbidly obese patients [12]. On the other hand, a comprehensive meta-analysis by Li et al. suggested that circulating betatrophin levels in T2D nonobese patients are higher than those in the nondiabetic control group [9]. Moreover, recent studies showed that plasma concentrations of metabolic markers, for example, betatrophin, as well as hepatocyte growth factor and nesfatin, are elevated in drug-naïve prediabetic and T2D patients in comparison to normoglycemic subjects [18, 19]. It has also been observed that serum betatrophin concentration positively correlated with HbA1c and fasting plasma glucose in the group of patients with diabetes, prediabetic state, and metabolic syndrome [19] and with fasting plasma glucose and HOMA-IR in T2D and nondiabetic subjects [20].

Some data indicate that serum betatrophin concentrations are positively connected with the level of C-peptide in obese nondiabetic subjects [15]. However, this observation was not present in T2D patients [15]. In other studies, serum betatrophin concentrations inversely correlated with the increment of C-peptide in the glucagon stimulation test in T2D patients [21]. This might indicate that the increase in serum betatrophin concentrations is a result of insulin resistance observed in obese nondiabetic subjects.

Apart from its role in glucose homeostasis, betatrophin has an impact on lipid metabolism. In experimental data, betatrophin suppressed adipose triglyceride lipase activity and increased TG accumulation in hepatocytes, adipocytes, and beta cells [22]. In the fasted state, similar serum TG levels in ANGPTL8^−/−^ mice and wild-type animals were noticed. However, lower serum TG concentrations in response to refeeding in mice lacking the betatrophin gene have been observed [23]. Moreover, starvation decreased betatrophin expression in BAT and WAT [6]. This could suggest that betatrophin influences lipid homeostasis in the refeeding state.

As it was mentioned earlier, betatrophin could be involved in glucose and lipid metabolism, which is often impaired in women with PCOS. Therefore, the aim of the present study was to investigate serum betatrophin concentrations after glucose load and its relationship with indirect indices of insulin resistance and insulin secretion in women with PCOS in comparison to the healthy controls.

## 2. Materials and Methods

### 2.1. Subjects

The study group consisted of 59 women—43 patients with PCOS and 16 healthy controls matched for BMI and age. The diagnosis of PCOS was made according to the 2003 Rotterdam ESHRE/ASRM PCOS Consensus Workshop Group diagnostic criteria [24]. We defined PCOS by the presence of at least two out of three criteria: clinical and/or biochemical hyperandrogenism, oligo/anovulation, and polycystic ovaries (>12 follicles measuring 2–9 mm in diameter or ovarian volume > 10 ml in at least one ovary) [24]. Women with PCOS were recruited from the outpatient clinic of the Department of Endocrinology, Diabetology and Internal Medicine, Medical University of Bialystok.

Clinical examination, anthropometric measurements, body fat assessment by bioelectrical impedance analysis, oral glucose tolerance test (OGTT) with 75 grams of glucose load, and ultrasonography of ovaries were performed as previously described [2]. Lipids, hormonal profiles (LH, FSH, total testosterone, and PRL), and serum betatrophin concentrations at the baseline and at 120 min of OGTT were estimated. Exclusion criteria included the following: morbid obesity, cardiovascular disease, and hyperlipidemia; other causes of irregular menstrual cycles and/or androgen excess (i.e., hyperprolactinemia, Cushing's syndrome, late-onset congenital adrenal hyperplasia, or other diseases of the thyroid, adrenal glands, pregnancy, and breastfeeding); type 1 or type 2 diabetes; chronic or acute infection (within the previous 30 days); any other serious medical problem, hormonal contraception, and/or antiandrogen therapy (within the previous 6 months); and the use of medications for obesity, hyperglycemia, dyslipidemia, or hypertension. All participants were nonsmokers. All analyses were carried out after an overnight fast. Studies were performed in the PCOS group 3–5 days after spontaneous menses or independently of cycle phase in the presence of amenorrhea and in regularly cycling women during the early follicular phase (3rd–5th day) of their menstrual cycle. All subjects gave written informed consent. The study protocol was approved by the Ethics Committee of Medical University of Bialystok, Poland, and was concordant with the Declaration of Helsinki.

### 2.2. Biochemical Analyses

Plasma glucose level was measured immediately by the enzymatic reference method with hexokinase (Cobas c111, Roche Diagnostic Ltd., Switzerland). Serum insulin concentration was assayed by immunoradiometric method (DIAsource ImmunoAssays S.A., Belgium). The minimum detectable concentration was 1 *μ*IU/ml and the intra-assay and interassay coefficients of variation (CVs) were below 2.2% and 6.5%, respectively. In this method, human and animal proinsulins present no cross-reactions.

Serum total cholesterol, HDL-cholesterol, and TG were assessed by the enzymatic methods using commercial kits produced by ANALCO-GBG, Poland. Serum LDL cholesterol was calculated according to Friedewald's formula.

Serum luteinizing hormone (LH) (sensitivity 0.2 mIU/ml; intra-assay CV 3.9%, interassay CV 3.4%), follicle-stimulating hormone (FSH) (sensitivity 0.1 mIU/ml; intra-assay CV 2.0%, interassay CV 4.4%), and serum prolactin (sensitivity 0.35 ng/ml; intra-assay CV 3.3%, interassay CV 4.5%) were measured by the immunoradiometric method (DIAsource ImmunoAssays S.A., Belgium). Serum total testosterone (sensitivity 0.05 ng/dl; intra-assay CV 4.6%, interassay CV 6.2%) was measured by the radioimmunoassay (DIAsource ImmunoAssays S.A., Belgium). Free-androgen index (FAI) was calculated as serum total testosterone (nmol/l) × 100/SHBG (nmol/l) ratio [25].

Serum betatrophin level was detected by enzyme-linked immunosorbent assays using commercially available reagents (USCN Life Science Inc., Wuhan, China); the detection range was 0.156–10 ng/ml and the sensitivity was less than 0.057 ng/ml. Intra- and interassay coefficients of variation were under 10% and 12%, respectively. Δ betatrophin was calculated as the difference in betatrophin between 0 min and 120 min of OGTT.

### 2.3. Calculations

Consider as following:

1. The homeostasis model assessment insulin resistance (HOMA-IR) was calculated according to the formula in [26]:

(1) $$\begin{array}{c} \frac{FPI\times FPG\;}{22.5}. \end{array}$$

(2) Whole-body insulin sensitivity was calculated by Matsuda index according to the formula in [27]:

(2) $$\begin{array}{c} \frac{10000}{\sqrt{\left(FPG\times FPI\right)\times \left(MPG\times MPI\right)}}. \end{array}$$

(3) Islet beta cell function was evaluated by homeostasis model assessment *β* cell function (HOMA-B) according to the formula in [26]:

(3) $$\begin{array}{c} \frac{20\times FPI}{FPG-3.5}, \end{array}$$

where FPI is fasting plasma insulin (*μ*IU/ml), FPG is fasting plasma glucose (mmol/l), MPG is mean plasma glucose during OGTT (mmol/l), and MPI is mean plasma insulin during OGTT (*μ*IU/ml).

### 2.4. Statistical Analysis

The statistics were performed with STATISTICA 10.0 software. The differences between the groups were evaluated with nonparametric Mann–Whitney *U* test. The relationships between variables were evaluated using Spearman's rank correlation and multiple linear regression analysis. The level of significance was accepted at *p* < 0.05.

## 3. Results

The clinical and biochemical characteristics of the studied groups are presented in Table 1. The PCOS and control women did not differ in age, anthropometric indices, plasma lipid concentrations, baseline plasma glucose concentrations, baseline serum insulin concentrations, HOMA-IR, Matsuda index, and HOMA-B (all *p* > 0.05) (Table 1). Women with PCOS had significantly elevated serum LH (*p* = 0.01), total testosterone concentrations (*p* = 0.009), and FAI (*p* = 0.0009) in comparison to the control group (Table 1).

In the PCOS group, higher plasma concentrations of glucose at 30′ (*p* = 0.03) and 60′ (*p* = 0.01) of OGTT, as well as higher serum insulin concentrations at 60′ (*p* = 0.02) of OGTT, in comparison to those in the control group were observed (Table 1).

Baseline serum betatrophin concentrations did not differ between the studied groups (*p* = 0.7). Glucose load resulted in an increase in serum betatrophin concentrations only in the control group (*p* = 0.02). In consequence, serum betatrophin concentrations at 120 min of OGTT were lower in women with PCOS in comparison to the control group (*p* = 0.02) (Table 1).

We found a positive correlation between baseline serum betatrophin concentrations and baseline serum insulin concentration (*r* = 0.42, *p* = 0.004), only in women with PCOS.

We found a negative relationship between Δ betatrophin and serum total testosterone concentration in the entire group (*r* = −0.32, *p* = 0.01).

**Figure 1:**
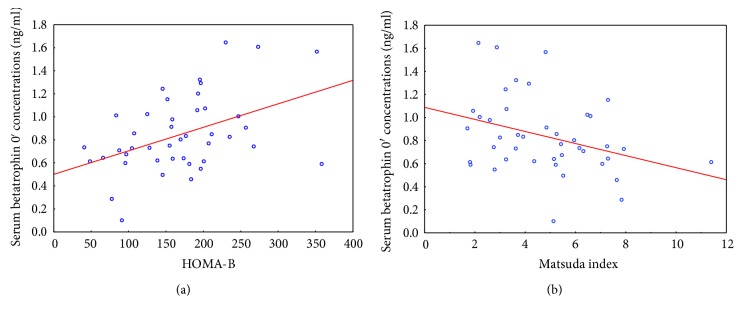
Correlation between serum betatrophin 0′ concentrations and HOMA-B (*r* = 0.38, *p* = 0.01) (a) and Matsuda index (*r* = −0.31, *p* = 0.004) (b) in women with PCOS.

We subsequently examined the relationship between baseline serum betatrophin and indirect indices of insulin resistance, as well as beta cell function in the studied groups. We observed a positive correlation of baseline betatrophin concentrations with HOMA-IR (*r* = 0.39, *p* = 0.008), a negative correlation with Matsuda index (*r* = −0.31, *p* = 0.004), and a positive relationship with HOMA-B (*r* = 0.38, *p* = 0.01) (Figure 1), only in women with PCOS.

In the next step, we created a linear regression model for betatrophin as a dependent variable. The model that best predicted betatrophin in patients with PCOS included HOMA-B as an independent variable (*p* = 0.001, *β* = 0.47). This model explained 22.3% of the variability in betatrophin concentrations (*R*^2^ = 0.223).

## 4. Discussion

The main observation of the present study is the different responses to glucose load in PCOS women in comparison to the healthy women. The glucose load did not cause any increase in serum betatrophin concentrations during OGTT in the PCOS group, contrary to what was observed in the control group. Additionally, we observed relations between serum betatrophin and indirect measures of insulin resistance, as well as HOMA-B, only in the PCOS group. We also found a negative relationship between Δ betatrophin during OGTT and serum total testosterone concentration in the entire group.

The important observation of the present study is the lack of an increase in serum betatrophin after glucose load during OGTT in the PCOS group, as it was observed in controls. There is no straightforward explanation of this phenomenon. In several murine studies, it was shown that fasting is a negative regulator of betatrophin mRNA expression in BAT, WAT, and liver [6, 7, 28]. In humans, one study examined the impact of meals on serum betatrophin concentrations. The authors evaluated 12 nonobese nondiabetic subjects and observed a statistically significant increase in serum betatrophin concentrations 2 hours after a mixed meal containing carbohydrate, protein, and fat [29]. This indicates that its concentrations in healthy subjects rise in response to food intake, as we did not notice the increase in serum betatrophin concentrations during OGTT in women with PCOS in comparison to the control group. Therefore, we can speculate that its disturbances in this group of patients could first appear in the postprandial state.

Other factors contributing to changes in betatrophin concentrations during glucose load still require identifying. In the present study, we observed an increased level of total testosterone in women with PCOS and an inverse correlation between Δ betatrophin and total testosterone levels in the entire group. Therefore, we can hypothesize that elevated total testosterone concentrations might influence serum betatrophin concentrations in response to glucose load during OGTT in the PCOS group. Similarly, Calan et al. showed that serum betatrophin is related to free-testosterone concentrations in PCOS [30]. Additionally, we can hypothesize that inappropriate response in releasing betatrophin after glucose load could lead to the development of glucose intolerance in women with PCOS.

Another observation of the present study is a finding that baseline serum betatrophin concentrations negatively correlated with indirect indices of insulin sensitivity, for example, Matsuda index, and positively with HOMA-IR and HOMA-B only in patients with PCOS. Moreover, in the linear regression analysis, we observed that baseline serum betatrophin concentration was strongly connected with HOMA-B. Therefore, we can assume that serum betatrophin concentrations depend on insulin secretion from beta cells in women with PCOS. As it was mentioned in the Introduction, it has been shown that serum betatrophin concentration positively correlated with C-peptide and HOMA-IR in obese nondiabetic subjects but not in T2D patients [15]. It could be possible that during the development of insulin resistance, betatrophin is not sufficient to compensate for an increased demand for insulin, as it was observed in T2D [14].

Our results are consistent with those of other researchers who showed a positive correlation between serum betatrophin concentration and HOMA-IR in women with PCOS [30] and in the group consisting of patients with T2D and prediabetes and in the control group [20]. Qu et al. also observed that fasting serum betatrophin concentration positively correlated with HOMA-IR only in women with PCOS [31], but did not estimate HOMA-B and Matsuda index. Additionally, Sahin et al. found that serum betatrophin level variability in the PCOS women was explained by homocysteine, HOMA-IR, and androstenedione levels [32]. Erol et al. reported an elevated serum betatrophin concentration in the PCOS women in comparison to the control group, although they did not evaluate HOMA-IR, Matsuda index, or HOMA-B [33].

Contradictory results have been recently reported by other researchers, who found a negative correlation between fasting serum betatrophin concentration and HOMA-IR [34–36]. Song et al. also observed a negative relationship between serum betatrophin concentration and HOMA-B in the PCOS women [34]. Different results obtained by the cited authors could be derived from differences in anthropometrical measurements of the studied groups. We examined Caucasian women with mean BMI 24.5 ± 3.1 kg/m^2^, whereas Song et al. studied Chinese women with mean BMI 25.4 ± 5.0 kg/m^2^ [34]. Additionally, our group of women with PCOS was younger (25.3 ± 4.5 yr versus 28.9 ± 4.9 yr). As betatrophin action could reflect a compensatory response in insulin resistant state and is proposed as a marker of insulin resistance, it is important to take these indices into consideration while comparing the results of different studies [20]. Moreover, we cannot exclude the influence of ethnic origin on serum betatrophin concentrations and different phenotypes of PCOS included in the quoted studies. However, the role of this hormone in the pathogenesis of insulin resistance in PCOS women is not established yet.

The main limitation of the present study is the relatively small sample size, especially in the control group.

## 5. Conclusions

On the basis of the obtained results, we concluded that serum concentrations of betatrophin are connected with insulin resistance and beta cell function and did not change after glucose load in women with PCOS.

## Figures and Tables

**Table 1:** Clinical and biochemical characteristics of the studied groups.

	Control group (*n* = 16)	PCOS (*n* = 43)
Age (years)	24.3 ± 4.1	25.3 ± 4.5
BMI (kg/m^2^)	22.8 ± 3.0	24.5 ± 3.1
Waist circumference (cm)	80.3 ± 7.9	85.2 ± 10.7
Hip circumference (cm)	99.7 ± 7.3	101.6 ± 8.4
FFM (kg)	45.5 ± 5.1	46.7 ± 6.0
FM (kg)	18.6 ± 6.6	22.6 ± 7.2^∗^
LDL cholesterol (mg/dl)	86.9 ± 20.4	100.5 ± 40.9
TG (mg/dl)	70.4 ± 17.1	78.7 ± 39.7
Glucose 0′ OGTT (mg/dl)	89 ± 6.8	88.2 ± 6.6
Glucose 30′ OGTT (mg/dl)	128.5 ± 24.6	145 ± 28.5^∗^
Glucose 60′ OGTT (mg/dl)	97 ± 22.7	123.5 ± 35.8^∗^
Glucose 120′ OGTT (mg/dl)	92 ± 15.6	95.7 ± 21.6
Insulin 0′ OGTT (*μ*IU/ml)	11.9 ± 5.5	11.5 ± 4.7
Insulin 30′ OGTT (*μ*IU/ml)	63.7 ± 28.5	89.3 ± 56.7
Insulin 60′ OGTT (*μ*IU/ml)	58.6 ± 49.2	84.4 ± 51.5^∗^
Insulin 120′ OGTT (*μ*IU/ml)	35.8 ± 16.9	56.4 ± 49.6
HOMA-IR	2.6 ± 1.2	2.5 ± 1.0
HOMA-B	175.1 ± 80.7	193 ± 186.3
Matsuda index	5.37 ± 1.7	4.8 ± 2.1
Follicle-stimulating hormone (IU/l)	5.8 ± 2.8	4.5 ± 1.4
Luteinizing hormone (IU/l)	3.7 ± 1.3	6.3 ± 3.9^∗^
Total testosterone (ng/ml)	0.5 ± 0.2	0.8 ± 0.4^∗^
SHBG (nmol/l)	48.0 ± 19.1	45.4 ± 22.6
FAI	4.3 ± 2.2	8.4 ± 5.9^∗^
Prolactin (ng/ml)	14.4 ± 6.8	15.2 ± 9.0
Betatrophin 0′ (ng/ml)	0.92 ± 0.35	0.85 ± 0.33
Betatrophin 120′ (ng/ml)	1.15 ± 0.42^#^	0.88 ± 0.32^∗^

Data are presented as mean ± SD. Differences between the groups are derived from nonparametric Mann–Whitney *U* test. BMI: body mass index; TG: triglycerides; FM: fat mass; FFM: fat-free mass; OGTT: oral glucose tolerance test; HOMA-IR: homeostasis model assessment insulin resistance; HOMA-B: homeostasis model assessment *β* cell function; FAI: free-androgen index; SHBG: sex hormone-binding globulin; ^∗^*p* < 0.05 in PCOS women versus control group; ^#^*p* < 0.05 versus the baseline state.

## Abbreviations

ANGPLT8: Atypical angiopoietin-like protein 8
BAT: Brown adipose tissue
BMI: Body mass index
CV: Coefficient of variation
FAI: Free-androgen index
FFM: Fat-free mass
FM: Fat mass
FSH: Follicle-stimulating hormone
HOMA-B: Homeostasis model assessment *β* cell function
HOMA-IR: Homeostasis model assessment insulin resistance
IR: Insulin resistance
LH: Luteinizing hormone
OGTT: Oral glucose tolerance test
PCOS: Polycystic ovary syndrome
RIFL: Refeeding-induced fat and liver protein
SHBG: Sex hormone-binding globulin
T1D: Type 1 diabetes mellitus
T2D: Type 2 diabetes mellitus
TG: Triglycerides
WAT: White adipose tissue.
